# Superfast Zincophilic Ion Conductor Enables Rapid Interfacial Desolvation Kinetics for Low‐Temperature Zinc Metal Batteries

**DOI:** 10.1002/advs.202401629

**Published:** 2024-05-09

**Authors:** Xiaomin Cheng, Yinze Zuo, Yongzheng Zhang, Xinyu Zhao, Lujie Jia, Jing Zhang, Xiang Li, Ziling Wu, Jian Wang, Hongzhen Lin

**Affiliations:** ^1^ *i*‐Lab & CAS Key Laboratory of Nanophotonic Materials and Devices Suzhou Institute of Nano‐Tech and Nano‐Bionics Chinese Academy of Sciences Suzhou 215123 P. R. China; ^2^ Institute of New Energy Materials and Engineering College of Materials Science and Engineering Fuzhou University Fuzhou 350108 P. R. China; ^3^ State Key Laboratory of Chemical Engineering East China University of Science and Technology Shanghai 200237 P. R. China; ^4^ School of Materials Science and Engineering Xi'an University of Technology Xi'an 710048 P. R. China; ^5^ Helmholtz Institute Ulm (HIU) D89081 Ulm Germany; ^6^ Karlsruhe Institute of Technology (KIT) D76021 Karlsruhe Germany

**Keywords:** desolvation kinetics, low‐temperature battery, rapid ion diffusion, zinc metal battery, zincophilic conductor

## Abstract

Low‐temperature rechargeable aqueous zinc metal batteries (AZMBs) as highly promising candidates for energy storage are largely hindered by huge desolvation energy barriers and depressive Zn^2+^ migration kinetics. In this work, a superfast zincophilic ion conductor of layered zinc silicate nanosheet (LZS) is constructed on a metallic Zn surface, as an artificial layer and ion diffusion accelerator. The experimental and simulation results reveal the zincophilic ability and layer structure of LZS not only promote the desolvation kinetics of [Zn(H_2_O)_6_]^2+^ but also accelerate the Zn^2+^ transport kinetics across the anode/electrolyte interface, guiding uniform Zn deposition. Benefiting from these features, the LZS‐modified Zn anodes showcase long‐time stability (over 3300 h) and high Coulombic efficiency with ≈99.8% at 2 mA cm^−2^, respectively. Even reducing the environment temperature down to 0 °C, ultralong cycling stability up to 3600 h and a distinguished rate performance are realized. Consequently, the assembled Zn@LZS//V_2_O_5‐x_ full cells deliver superior cyclic stability (344.5 mAh g^−1^ after 200 cycles at 1 A g^−1^) and rate capability (285.3 mAh g^−1^ at 10 A g^−1^) together with a low self‐discharge rate, highlighting the bright future of low‐temperature AZMBs.

## Graphical abstract

A superfast ionic conductor for achieving fast Zn^2+^ desolvation and efficient ion transport is pioneered, which accelerates the Zn^2+^ transport kinetics and guides uniform Zn deposition, as revealed by theoretical calculations and experimental characterizations. The Zn@LZS symmetric cell exhibits ultralong cycling stability of up to 3600 h under 0 °C.

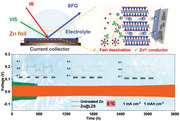

## 1 Introduction

Rechargeable aqueous zinc metal batteries (AZMBs), as a high‐safe and low‐cost electrochemical energy storage technology, have recently gained appealing attention owing to the specific capacity (820 mAh g^−1^) and moderate redox potential (−0.76 V vs. SHE) of zinc anode.^[^ ^1^ ^]^ However, the hydrogen evolution reaction (HER) and Zn corrosion as well as rampant zinc dendrite growth lead to unsatisfactory cyclic reversibility and Coulombic efficiency (CE).^[^ ^2^ ^]^ The performance of Zn anode is further degenerated especially under high current density or low‐temperature surroundings, ultimately bringing about internal short and battery failure.^[^ ^3^ ^]^ In essence, the above problems result from the imperfect Zn ion behaviors at the electrode/electrolyte interface, which suffers from the large desolvation energy barrier of [Zn(H_2_O)_6_]^2+^ cluster and the depressive Zn^2+^ migration kinetics.^[^ ^4^ ^]^ Therefore, to realize the eventual practical application of AZMBs, the electrode/electrolyte interface must be rationally designed to strengthen the desolvation capability and ion diffusion kinetics.

To date, versatile endeavors have been taken to stabilize the electrode/electrolyte interface by modulating the solvation sheath of [Zn(H_2_O)_6_]^2+^ cluster. Electrolyte optimization is a popular strategy for protecting the Zn anode, which effectively attenuates the interaction between electrons of Zn metal and active water.^[^ ^5^ ^]^ Although electrolyte engineering can change the solvation shell and regulate the coordination environment of Zn^2+^ via a crowed strategy, it sacrifices the deposition overpotential of zinc and impairs the ion conductivity accompanied by high costs.^[^ ^6^ ^]^ Another feasible strategy is to construct versatile artificial layers on Zn anode, such as covalent organic framework^[^ ^7^ ^]^ and molecular sieve,^[^ ^8^ ^]^ which not only functionalize as physical barriers to physically separate H_2_O from Zn anode via sieving the Zn^2+^‐solvents complex by suitable pore size, but also average the plating/stripping behavior of Zn^2+^. However, the sieving effect to dissociate H_2_O from [Zn(H_2_O)_6_]^2+^ only depends on the relative size of pores and [Zn(H_2_O)_6_]^2+^ cluster, which does not fundamentally reduce the desolvation energy barrier.^[^ ^8^, ^9^ ^]^ Therefore, more zincophilic adsorption sites with rapid ion exchange capability need to be designed to accelerate the desolvation kinetics at the electrode/electrolyte interface.

Generally, the plating process of zinc atom (Zn^0^) contains multi‐steps, including the desolvation of [Zn(H_2_O)_6_]^2+^, the diffusion of Zn^2+^, the formation of Zn^0^ after getting electrons, and the migration of the formed Zn^0^.^[^ ^10^ ^]^ Apart from reducing the desolvation energy barrier, accelerating the Zn^2+^ diffusion kinetics with even Zn^2+^ flux is also a vital requirement to achieve uniform and consecutive nucleation, giving dendrite‐free Zn electroplating behavior. Therefore, an ideal artificial solid electrolyte interphase (SEI) should satisfy the functionality of rapid desolvation and fast Zn^2+^ conduction at the electrolyte/anode interface.^[^ ^11^ ^]^ Impressively, layered clays (kaolin and montmorillonite) have raised much concern about Zn anode due to their effortless regulated ionophilic sites, characteristic layered structure, and fast ion transfer channels.^[^ ^12^ ^]^ Meanwhile, the diffusion pathway with sufficient tunnels has a relationship with fast Zn^2+^ transport and Zn^2+^ flux regulation.^[^ ^13^ ^]^ However, endeavors of layered clay‐structure materials only focus on the Zn^2+^ transfer tunnels, and the effect of promoting desolvation is completely unknown. Therefore, it is necessary to construct an artificial SEI layer with the capability in accelerating desolvation of [Zn(H_2_O)_6_]^2+^ and providing rapid diffusion of naked Zn^2+^ across the electrolyte/electrode interface.

**Figure 1:**
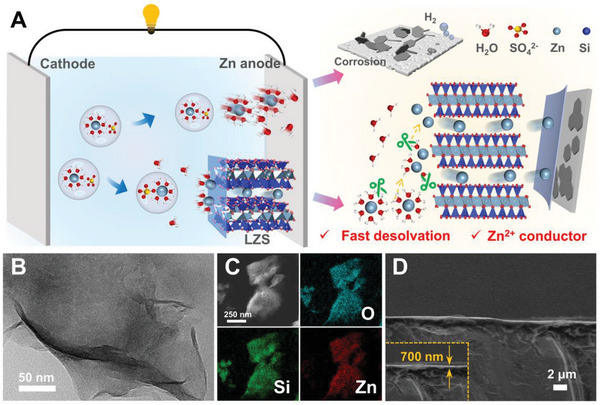
A) Schematic illustration of anode/electrolyte interface in accelerating desolvation and promoting Zn^2+^ diffusion kinetics. B) TEM image of the synthesized LZS and C) corresponding EDS elemental mappings. D) Cross‐sectional SEM image of Zn@LZS anode.

Herein, the layered zinc silicate nanosheets (LZS) with abundant zincophilic sites and transport channels to guarantee superfast ion diffusion are developed as the artificial SEI layer for Zn anode (**Figure** **1**A), regulating the desolvation kinetics and Zn^2+^ transfer behavior. The zincophilic ability of LZS significantly changes the solvation structure of [Zn(H_2_O)_6_]^2+^ at the anode/electrolyte interface and reduces the desolvation energy barrier, as revealed by theoretical simulation and experimental measurement, which is conducive to release more free Zn^2+^. Besides, the fleet and continuous Zn^2+^ transference can be realized by abundant nano‐scale diffusive paths. Consequently, the designed LZS layer improves the diffusion kinetics of Zn^2+^ toward the Zn anode, averaging the Zn^2+^ flux around the Zn surface. The assembled LZS‐decorated Zn‐based symmetric cell delivers an excellent cycling performance for over 3300 h with an ultralow overpotential (23.2 mV) at 0.5 mA cm^−2^ and an outstanding Zn plating/stripping reversibility of 99.8%. Even under low‐temperature environments, long‐time cycling stability (>3600 h) and rate performance with lower voltage hysteresis (48.2 mV) are also obtained. This superior electrochemical performance is further verified by assembling Zn@LZS//V_2_O_5‐x_ full cells, demonstrating its great practical application prospects.

## 2 Results and Discussion

Layered zinc silicate (LZS) nanosheets were prepared through the hydrothermal method. As illustrated in Figure 1A, the as‐prepared LZS possesses unique Zn–O bridges connected to Si–O tetrahedral layers, forming a repeating unit of the sandwich‐like layer structure.^[^ ^14^ ^]^ Its affluent zincophilic sites provide the impetus for the dissociation of [Zn(H_2_O)_6_]^2+^, and then the rapid and homogeneous migration of free Zn^2+^ is realized by sufficient nanoscale channels. The transmission electron microscopy (TEM) image of LZS in Figure 1B unfolds a nanosheet‐like morphology, where the Zn, Si, and O elements are evenly distributed in the zinc silicate nanosheets (Figure 1C). After the spraying process, it can be seen from the cross‐sectional scanning electron microscope (SEM) image that the thickness of the LZS layer is ≈700 nm, which is suitable to guarantee the rapid desolvation and the fast ion transport (Figure 1D; Figure S1, Supporting Information). These nanosheets have the trend to form a stacked structure with face‐to‐face mode, which can provide multiple and evenly distributed zincophilic sites, enhancing the desolvation kinetics and producing more naked Zn^2+^. N_2_ adsorption‐desorption isotherm and corresponding pore size distribution (Figure S2, Supporting Information) reveal the co‐existence of micro‐ and meso‐pores concentrated at ≈1.2 and 3.5 nm, suggesting the relatively concentrated pore structure of LZS. The atomic ratio of Zn and Si in the nanosheets is determined to be 0.75. Combined with the X‐ray diffraction (XRD) pattern in Figure S2C (Supporting Information), the crystalline structure is consistent with Zn_3_Si_4_O_10_(OH)_2_ •H_2_O, a clay‐type silicate.^[^ ^14^, ^15^ ^]^ In addition, a strong peak at 7.16° emerges, corresponding to a layer space of 1.23 nm, which is exactly matched with the TEM and pore structure results.

**Figure 2:**
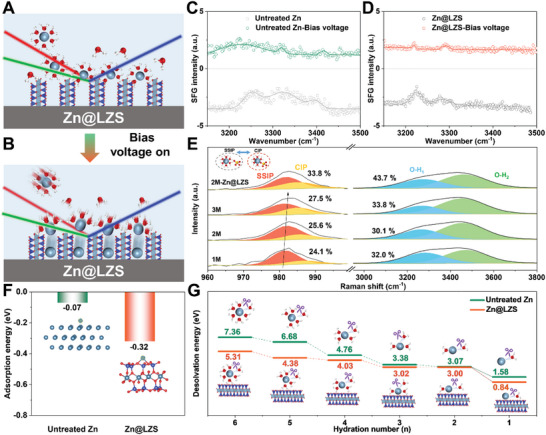
A, B) Schematic illustration of Zn^2+^ dissociation with/without the bias voltage of −20 mV. SFG‐intensity changes of O─H region in the C) untreated Zn/electrolyte and D) Zn@LZS/electrolyte interface. E) Raman spectra of *v*‐SO_4_^2−^ band and O─H band. F) Adsorption energies and the corresponding absorbed models of Zn atom on the untreated Zn and LZS layer. G) Comparison of energy barriers during the step‐by‐step desolvation process of [Zn(H_2_O)_n_]^2+^.

[Zn(H_2_O)_6_]^2+^ at the electrolyte/electrode interface needs to be sieved before being reduced and deposited, which is urgently desirable for fast ion transport.^[^ ^16^ ^]^ In situ, sum frequency generation (SFG) spectroscopy is frequently applied for probing the molecular vibrations at the electrode/electrolyte interface due to its interface‐sensitive.^[^ ^17^ ^]^ **Figures** **2**A,B and S5 (Supporting Information) depict the evolution process of the [Zn(H_2_O)_6_]^2+^ at the interface with/without bias voltage. Apparent solvent peaks of the O─H bond are observed at ≈3240, 3325, and 3400 cm^−1^ (Figure 2C,D), and O─H band strength at the Zn@LZS/electrolyte interface is smaller than that at the untreated Zn/electrolyte interface, indicating the preliminary dissociation of [Zn(H_2_O)_6_]^2+^. When a 20 mV bias voltage is applied, the dynamic dissociation process of Zn^2+^‐solvents at the interface is expedited. Obviously, both the intensity of O─H vibrations with/without LZS is reduced, where the intensity at the Zn@LZS/electrolyte interface declines significantly, showing the superiority of LZS in accelerating the dissociation of [Zn(H_2_O)_6_]^2+^. In addition, Raman spectroscopy was performed to evaluate the solvation structure at the anode/electrolyte interface. Generally, two types of ion‐pair species (solvent‐separated ion pairs (SSIP, [Zn^2+^(H_2_O)_6_·OSO_3_^2−^]) and contact ion pairs (CIP, [Zn^2+^(H_2_O)_5_·OSO_3_^2−^]) can be separated according to Eigen‐Tamm (ET) mechanism.^[^ ^16^, ^18^ ^]^ As depicted in Figure 2E, with an increasing concentration of ZnSO_4_, the percentage of CIP progressively increases, indicating the significant enhancement of the coupling degree between Zn^2+^ and SO_4_^2−^. Especially, at the Zn@LZS/electrolyte interface, the percentage of CIP is 33.8%, which is higher than that of 25.6% at the untreated Zn/electrolyte interface, meaning the formation of a closer [Zn^2+^SO_4_^2−^] ion association with the LZS layer. The variation trend of O─H stretch vibration (3000–3800 cm^−1^) can be divided into two peaks, namely robust hydrogen bond (O─H_1_) and weaker hydrogen bond (O─H_2_).^[^ ^16^, ^18^, ^19^ ^]^ Similarly, a suppressed stretch of O─H_1_ appears with the concentration of ZnSO_4_ increases, and the distribution of O─H_1_ at the Zn@LZS/electrolyte interface is smaller than the distribution of O─H_1_ at the untreated Zn/electrolyte interface, affirming the changed solvation structure of hydrated Zn^2+^ at the interface due to the LZS layer. The activation energy (E_a_) was quantified to describe the desolvation kinetics (Figure S7, Supporting Information), where the Zn@LZS delivered a smaller E_a_ (21.0 kJ mol^−1^) than that of untreated Zn (26.2 kJ mol^−1^), propelling the desolvation kinetics for releasing more free Zn^2+^.^[^ ^20^ ^]^

The desolvation process of [Zn(H_2_O)_6_]^2+^ at the anode/electrolyte interface determines subsequent ion diffusion kinetics.^[^ ^17^, ^21^ ^]^ Density functional theory (DFT) calculations were performed to explain the enhanced desolvation and Zn^2+^ diffusion kinetics by the LZS layer. As shown in Figure 2F, a higher Zn atom adsorption preference (−0.32 eV) on the LZS layer is discovered compared with untreated Zn (−0.07 eV), the stronger adsorption between the Zn atom and the LZS layer can effectively boost the desolvation of [Zn(H_2_O)_6_]^2+^. The step‐by‐step desolvation processes of [Zn(H_2_O)_n_]^2+^ are shown in Figure 2G. It is obvious that the dissociation of [Zn(H_2_O)_n_]^2+^ at the Zn@LZS/electrolyte interface requires less energy than that at the untreated Zn/electrolyte interface in each desolvation. For example, [Zn(H_2_O)_6_]^2+^ at the untreated Zn/electrolyte interface requires 7.36 eV to release one water, while only 5.31 eV is needed at Zn@LZS/electrolyte interface, further proofing the advantage of the LZS layer in desolvation process. Such a low energy barrier of desolvation contributes to  the accelerated Zn^2+^ diffusion kinetics.

**Figure 3:**
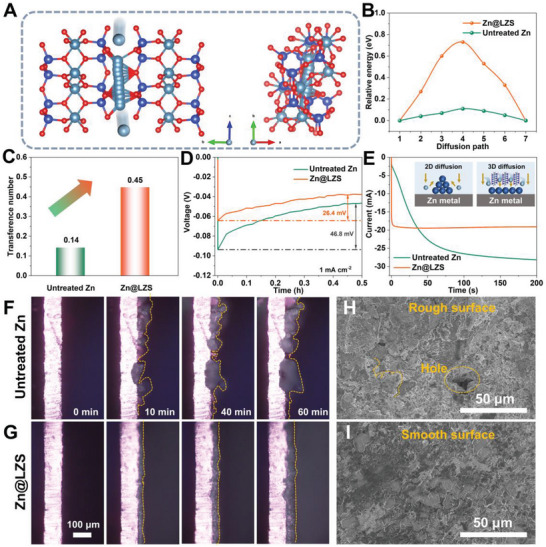
A) The possible migration pathway on LZS and B) calculated migration energy barriers of Zn^2+^ along the c‐axis. C) Zn^2+^ transference number of untreated Zn and Zn@LZS anode. D) Nucleation overpotentials of the untreated Zn and Zn@LZS. E) Chronoamperometry curves of untreated Zn and Zn@LZS anode at −150 mV overpotential. In situ optical microscopy observation of Zn plating on F) untreated Zn and G) Zn@LZS. Deposition morphology of H) untreated Zn and I) Zn@LZS anode after 50 cycles with high‐power ultrasonication at 1 mA cm^−2^.

**Figure** **3**A,B display a possible Zn^2+^ diffusion pathway and corresponding energy barriers. The calculated energy barrier on the LZS is 0.72 eV, higher than that of the untreated Zn surface (0.11 eV). An ultra‐low diffusion barrier can cause uncontrolled 2D diffusion, generating local atomic clusters and inducing uneven nucleation.^[^ ^22^ ^]^ Considering the migration barrier of multivalent‐cation is generally high, the energy barrier of 0.72 eV is a relatively low value compared to other published papers.^[^ ^12^, ^23^ ^]^ These calculated results indicate that the effortless desolvation, fast, and homogeneous Zn^2+^ transport occur within the LZS layer. The desolvation ability and Zn^2+^ diffusion kinetics are further appraised by analyzing ionic conductivity (σ) and Zn^2+^ transference number ($t_{Zn^{2+}}$). The detailed data analysis of σ and $t_{Zn^{2+}}$ are presented in Figures S9 and S10 (Supporting Information). The σ value of the LZS layer is calculated to be 2.16 mS cm^−1^, which is comparable to or even better than the reported coatings (Table S2, Supporting Information), justifying the advantages of the artificial LZS layer in enhancing the rapid migration of desolvated Zn^2+^.^[^ ^12c^ ^]^ Meanwhile, the LZS‐coated Zn displays a higher $t_{Zn^{2+}}$ of 0.45, that is, 3.2 times greater than that for untreated Zn (Figure 3C). On the one hand, the enhanced $t_{Zn^{2+}}$ and the higher σ are attributed to the fast dissociation of Zn^2+^‐solvation structure and the release of numerous naked Zn^2+^, providing sufficient ions for subsequent diffusion and nucleation. On the other hand, the LZS layer with abundant nano‐scale diffusive paths can act as a Zn^2+^ conductor, accelerating the lateral diffusion of free Zn^2+^ for smooth plating. Ultimately, the fast Zn^2+^‐transfer kinetics is successfully realized by the synergy of enhanced desolvation process and selective pathway for Zn^2+^ diffusion.

Benefiting from the rapid desolvation and the enhanced Zn^2+^ transport kinetics, Zn nucleation and growth behaviors are significantly optimized. As demonstrated in Figure 3D, Zn@LZS exhibits a lower overpotential of 26.4 mV compared with untreated Zn of 46.8 mV. Additionally, the stronger current intensities of Zn@LZS than those of untreated Zn elucidate more free Zn^2+^ coupled with electrons (Figure S11, Supporting Information).^[^ ^24^ ^]^ In Figure 3E, untreated Zn exhibits a continuous current rise throughout 200 s, justifying the whole process is dominated by 2D diffusion due to the irregular nucleation resulting from inhomogeneous Zn^2+^ flow and locally concentrated diffusion. Differing from untreated Zn, Zn@LZS symmetric cell displays a transient 2D diffusion, followed by a constant and stable 3D diffusion, meaning that a uniformly and rapidly regulated Zn^2+^ flux is acquired due to the fast desolvation and efficient ion transport pathways, ultimately resulting in even Zn deposition.^[^ ^25^ ^]^

To further evaluate the modulated Zn growth behavior enabled by the LZS layer, an in situ optical microscope and SEM were employed to directly visualize the deposition morphology evolution of Zn. As shown in Figure 3F, randomly cliff‐like Zn aggregates appear along the edge of Zn foil only after 2 min. These visible protrusions continuously grow up over time and evolve into an uneven surface.^[^ ^26^ ^]^ In comparison, a dense and flat surface without Zn dendrite is achieved on Zn@LZS anode during the whole process, indicating the impact of rapid desolvation and fast interfacial Zn^2+^ transport kinetics on the Zn deposition behavior, which guides uniform Zn deposition and suppresses Zn dendrite growth. The same difference is also characterized by SEM images (Figure 3G,H). After 50 cycles, numerous rough dendritic microstructure and corrosion byproducts with serious holes are stacked on untreated Zn surface. While the Zn@LZS anode presents a compact and smooth surface without dendrites, further confirming the positive effect of LZS layer.

**Figure 4:**
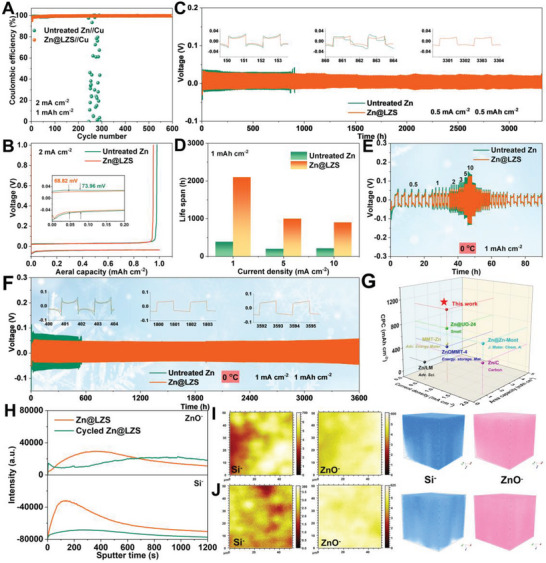
A) Coulombic efficiency and corresponding B) voltage/capacity plots of untreated Zn//Cu and Zn@LZS//Cu at 2 mA cm^−2^. C) Long‐term galvanostatic charge/discharge curves at 0.5 mA cm^−2^ for 0.5 mAh cm^−2^. D) The summary of long‐term galvanostatic charge/discharge performance at different current densities. E) Rate performance and F) long‐term galvanostatic charge/discharge curves at 0 °C. G) The comparison of calculated cumulative plated capacity (CPC) with the other literatures. H) Si^−^ and ZnO^−^ depth profiles of Zn@LZS and cycled Zn@LZS. The top‐view surface mapping and 3D reconstructions of Si^−^ and ZnO^−^ species in I) Zn@LZS and J) cycled Zn@LZS at 1 mA cm^−2^ via TOF‐SIMS.

Coulombic efficiency was performed to evaluate the role of the LZS layer on the plating/stripping reversibility. As shown in **Figure** **4**A, the Zn//Cu half cell appears to fluctuate Coulombic efficiency ≈80 cycles and fails to operate after less than 235 cycles, attributing to the continuous growth of Zn dendrites during cycling. In comparison, the Coulombic efficiency of the Zn@LZS//Cu half cell quickly surpasses 99.8% and holds extremely steady over 600 cycles, justifying excellent Zn plating/stripping reversibility with LZS layer. Moreover, the overpotential of the Zn@LZS//Cu cell is 68.8 mV, smaller than that of untreated Zn//Cu (74 mV), further confirming the enhanced Zn^2+^ transfer kinetics derived from the reduced desolvation barrier and depressed diffusion barrier (Figure 4B). The enhanced desolvation kinetics of [Zn(H_2_O)_6_]^2+^ and the promoted Zn^2+^ diffusion are also well reflected in symmetirc cells (Figure 4C,D). Cycled at 0.5 mA cm^−2^ for 0.5 mAh cm^−2^, the untreated Zn symmetric cell shows a high voltage hysteresis (30.0 mV) and fluctuation with a sudden voltage drop after 860 h due to the short‐circuit caused by dendrite growth. By contrast, a long cycling life of 3300 h and ultralow voltage hysteresis of 23.2 mV with negligible voltage oscillation are enabled by Zn@LZS symmetric cells under identical conditions.

As the current density extended to 1 mA cm^−2^ (1 mAh cm^−2^), the Zn@LZS symmetric cell operates steadily over 2100 h with a smaller voltage fluctuation (Figure 4D; Figure S13, Supporting Information). Even enhanced to 5.0 and 10.0 mA cm^−2^ (1 mAh cm^−2^), a similar comparative trend is still detected (Figure S13B,C, Supporting Information). The symmetrical cells with Zn@LZS anodes show a prolonged lifespan of 370 and 240 h even under the super high areal capacity of severe depth of discharge (DOD) condition of 34.0% and 51.2%, respectively, confirming the synergistic effect of rapid desolvation and fast Zn^2+^ diffusion by LZS layer (Figure S14, Supporting Information). The excellent symmetric cell performance of Zn@LZS is superior to most of the modified Zn anodes reported so far (Figure 4E‐4G). The effectiveness of the LZS layers also enables Zn anode to exhibit outstanding low‐temperature performance. Under harsh conditions (0 °C, Figure 4E), the Zn@LZS symmetric cell shows excellent rate performance. Specifically, with increasing the current density from 0.5 to 10.0 mA cm^−2^, the voltage hysteresis of 38.7, 45.1, 55.5, 64.3, 83.5, and 124.1 mV are detected, much lower than that of symmetric cell with untreated Zn. The impressive low‐temperature cycling stability of Zn@LZS symmetric cell is also exhibited in Figure 4F, which shows the long‐time cycling stability (>3600 h) and lower voltage hysteresis (48.2  vs. 69.3 mV). The prolonged lifespan and lower voltage hysteresis can be attributed to the decreased desolvation barrier and accelerated Zn^2+^ diffusion kinetics induced by LZS layer.

In addition, the time‐of‐flight secondary ion mass spectrometry (TOF‐SIMS) was conducted to detect the stability of the LZS coating during the plating/stripping processes. As shown in Figure 4H, the signal of Si^−^ increases first and then decreases with the extension of sputtering time, indicating the presence of LZS layer on the surface of Zn foil. After being cycled, a strong Si^−^ signal is still obvious, meaning the stability of the LZS coating. The top‐view surface mapping and 3D render overlay of Si^−^ in cycled Zn@LZS (Figure 4J) also show the uniform distribution of Si element, further verifying the stability of the LZS coating during the cycle. The robust structure and the flexibility of LZS nanoscale interlayer can buffer the stress caused by Zn^2+^ continuous diffusion to ensure the stability of the LZS during repeated stripping and plating processes. Moreover, the uniform distribution of ZnO^−^ on the surface of Zn@LZS also confirms the uniform deposition of zinc with the help of LZS layer.

**Figure 5:**
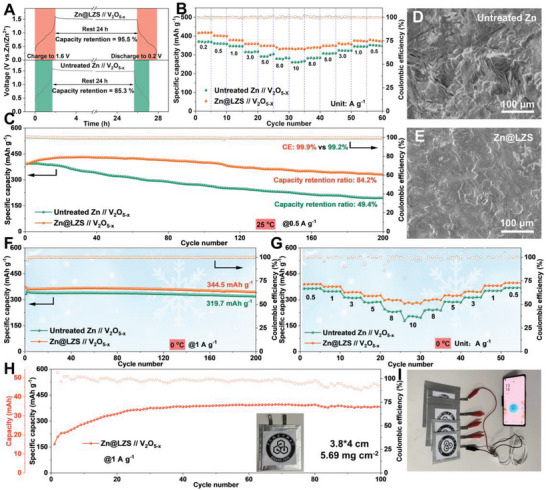
A) Self‐discharge behavior of full cells under resting for 24 h. B) The rate and C) cycling performance of full cells at 25 °C. SEM of cycled D) untreated Zn anode and E) Zn@LZS anode. F) Cycling and g) rate performance of full cells at 0 °C. H) Cycling performance of Zn@LZS//V_2_O_5‐x_ pouch cell at 1 A g^−1^. I) Photographs of pouch cells for powering the mobile phone.

Full cells employed with commercial V_2_O_5‐x_ cathode were assembled to evaluate the suitability of Zn@LZS anodes. As shown in **Figure** **5**A, Zn@LZS//V_2_O_5‐x_ cell maintains 95.5% of its initial capacity after 24 h resting compared to 85.3% of untreated Zn//V_2_O_5‐x_ cell, suggesting the inhibitation of LZS toward side reaction. In Figure 5B, the Zn@LZS electrode presents a specific capacity of 419.4 mAh g^−1^ at 0.2 A g^−1^, which preserves 332.4 mAh g^−1^ when the current density soars to 10 A g^−1^, whereas the capacity of the untreated Zn//V_2_O_5‐x_ decreases to 261.0 mAh g^−1^. The excellent rate capability of Zn@LZS//V_2_O_5‐x_ cell is likely to be due primarily to the rapid desolvation of [Zn(H_2_O)_6_]^2+^ and fast Zn^2+^ transport kinetics enabled by LZS layer. The long‐term cycling stability curves are displayed in Figure 5C and Figure S18 (Supporting Information). The cell with Zn@LZS electrode delivers a high specific capacity of 432.1 mAh g^−1^ and maintains at 343.5 mAh g^−1^ with a high Coulombic efficiency of 99.9% at 0.5 A g^−1^ after 200 cycles, which is comparable with the previous papers in some key electrochemical parameters (Table S3, Supporting Information). In contrast, a specific capacity of only 204.4 mAh g^−1^ with a low Coulombic efficiency of 99.2% is left for untreated Zn//V_2_O_5‐x_ cells. In addition, the performance of cells under the limited Zn anode (negative/positive capacity (N/P) = 15) and controlled electrolyte (electrolyte/capacity (E/C) = 30 µL mAh^−1^) were conducted. The capacity of Zn//V_2_O_5‐x_ cell decays rapidly during cycles at 0.5 A g^−1^, while the Zn@LZS//V_2_O_5‐x_ cell can stabilize for 50 cycles with high specific capacity, illustrating that the LZS layer has an obvious effect on stabilizing Zn anode. Benefiting from the effortless desolvation and ultrafast ion transport inside LZS‐induced full cells, the cycled Zn@LZS anode demonstrates a homogeneous and dense Zn deposition layer without dendrites, which is totally different from that of untreated Zn (Figure 5D,E). Impressively, under low‐temperature surroundings of 0 °C, the Zn@LZS//V_2_O_5‐x_ cell delivers a high specific capacity of 344.5 mAh g^−1^ with a capacity retention of 94.4% at 1 A g^−1^ after 200 cycles. In contrast, a low specific capacity of 319.7 mAh g^−1^ is exhibited with untreated Zn electrodes. More importantly, the Zn@LZS electrode outputs higher specific capacities than that of untreated Zn electrodes with the increase of current densities. Switching back to 0.5 A g^−1^, the cell with Zn@LZS electrode can be restored to its original specific capacity, meaning high reversibility and stability. The splendid low‐temperature stability and rate capability coincide with the functions of the LZS layer in accelerating the desolvation and increasing Zn^2+^‐transfer kinetics. In addition, the assembled Zn@LZS//MnO_2_ cells have better electrochemical performance than that of the Zn//MnO_2_ cells (Figure S20, Supporting Information). And the assembled large‐sized pouch cell renders a high capacity of 367 mAh g^−1^ after 100 cycles at 1 A g^−1^, which successfully powers the phone, endowing huge application potential of LZS artificial layer for AZMBs.

## 3 Conclusion

In summary, a layered zinc silicate with abundant zincophilic sites and sufficient diffusion channels is designed, which served as Zn^2+^ modulation layer to realize homogeneous and dense Zn plating behaviors. On the one hand, both theoretical simulation and in situ SFG reveal the enhanced desolvation of [Zn(H_2_O)_6_]^2+^ at the anode/electrolyte interface due to the zincophilic ability, which can release more free Zn^2+^. On the other hand, the abundant nano‐scale diffusive paths are conducive to speeding up the Zn^2+^ transport kinetics, enabling the uniform Zn^2+^ flux and achieving a dendrite‐free cycled surface. Consequently, the Zn@LZS anode enables a low voltage polarization of 23.2 mV, a long lifespan of 3300 h, and a high Coulombic efficiency of 99.8%. Moreover, an ultralong cycling stability over 3600 h with low voltage hysteresis and a distinguished rate performance can be realized even reducing the environment temperature to 0 °C. The excellent low‐temperature stability (344.5 mAh g^−1^ after 200 cycles at 1 A g^−1^) and rate capability are also obtained by assembling Zn@LZS//V_2_O_5‐x_ full cells. This easy‐to‐implement and low‐cost strategy of adjusting ion transport dynamics to promote uniform zinc plating offers broad prospects for the development of high‐performance aqueous zinc metal batteries.

## Conflict of Interest

The authors declare no conflict of interest.

## Supporting information

Supporting Information

## Data Availability

The data that support the findings of this study are available from the corresponding author upon reasonable request.

## References

[1] a) Y. Zhang , Z. Cao , S. Liu , Z. Du , Y. Cui , J. Gu , Y. Shi , B. Li , S. Yang , Adv. Energy Mater. 2022, 12, 2013979;

[2] C. Kao , C. Ye , J. Hao , J. Shan , H. Li , S. Qiao , ACS Nano 2023, 17, 3948. 36744842 10.1021/acsnano.2c12587

[3] a) M. Chen , S. Xie , X. Zhao , W. Zhou , Y. Li , J. Zhang , Z. Chen , D. Chao , Energy Storage Mater. 2022, 51, 683;

[4] a) J. Cao , D. Zhang , X. Zhang , Z. Zeng , J. Qin , Y. Huang , Energy Environ. Sci. 2022, 15, 499;

[5] a) W. Xu , J. Li , X. Liao , L. Zhang , X. Zhang , C. Liu , K. Amine , K. Zhao , J. Lu , J. Am. Chem. Soc. 2023, 145, 22456; 37802095 10.1021/jacs.3c06523

[6] S. Bai , Z. Huang , G. Liang , R. Yang , D. Liu , W. Wen , X. Jin , C. Zhi , X. Wang , Adv. Sci. 2024, 11, 2304549. 10.1002/advs.202304549 PMC10811481 38009799

[7] a) S. Park , I. Kristanto , G. Y. Jung , D. B. Ahn , K. Jeong , S. K. Kwak , S. Y. Lee , Chem. Sci. 2020, 11, 11692; 34123199 10.1039/d0sc02785e PMC8162792

[8] a) J. Zhu , Z. Bie , X. Cai , Z. Jiao , Z. Wang , J. Tao , W. Song , H. J. Fan , Adv. Mater. 2022, 34, 2207209; 10.1002/adma.202207209 36065756

[9] H. Yang , Y. Qiao , Z. Chang , H. Deng , X. Zhu , R. Zhu , Z. Xiong , P. He , H. Zhou , Adv. Mater. 2021, 33, 2102415. 10.1002/adma.202102415 34338385

[10] M. Xue , J. Bai , M. Wu , Q. He , Q. Zhang , L. Chen , Energy Storage Mater. 2023, 62, 102940.

[11] a) H. Sun , Y. Huyan , N. Li , D. Lei , H. Liu , W. Hua , C. Wei , F. Kang , J. G. Wang , Nano Lett. 2023, 23, 1726; 36794942 10.1021/acs.nanolett.2c04410

[12] a) C. Deng , X. Xie , J. Han , Y. Tang , J. Gao , C. Liu , X. Shi , J. Zhou , S. Liang , Adv. Funct. Mater. 2020, 30, 2000599;

[13] H. Peng , Y. Fang , J. Wang , P. Ruan , Y. Tang , B. Lu , X. Cao , S. Liang , J. Zhou , Matter 2022, 5, 4363.

[14] J. Qu , C.‐Y. Cao , Y.‐L. Hong , C.‐Q. Chen , P.‐P. Zhu , W.‐G. Song , Z.‐Y. Wu , J. Mater. Chem. 2012, 22, 3562.

[15] H. Liu , C. Yang , M. Han , C. Yu , X. Li , Z. Yu , J. Qu , Angew. Chem., Int. Ed. 2023, 62, 202217458. 10.1002/anie.202217458 36640120

[16] H. Liu , Z. Xin , B. Cao , Z. Xu , B. Xu , Q. Zhu , J. L. Yang , B. Zhang , H. J. Fan , Adv. Funct. Mater. 2024, 34, 2309840.

[17] a) J. Wang , H. Hu , J. Zhang , L. Li , L. Jia , Q. Guan , H. Hu , H. Liu , Y. Jia , Q. Zhuang , S. Cheng , M. Huang , H. Lin , Energy Storage Mater. 2022, 52, 210;

[18] H. Yang , Z. Chang , Y. Qiao , H. Deng , X. Mu , P. He , H. Zhou , Angew. Chem. Int. Ed. 2020, 59, 9377. 10.1002/anie.202001844 32202034

[19] W. Wang , S. Chen , X. Liao , R. Huang , F. Wang , J. Chen , Y. Wang , F. Wang , H. Wang , Nat. Commun. 2023, 14, 5443. 37673895 10.1038/s41467-023-41276-9 PMC10482877

[20] Q. Zong , B. Lv , C. Liu , Y. Yu , Q. Kang , D. Li , Z. Zhu , D. Tao , J. Zhang , J. Wang , Q. Zhang , G. Cao , ACS Energy Lett. 2023, 8, 2886.

[21] a) X. Li , Y. Zuo , Y. Zhang , J. Wang , Y. Wang , H. Yu , L. Zhan , L. Ling , Z. Du , S. Yang , Adv. Energy Mater. 2024, 14, 2303389;

[22] Z. Yang , C. Lv , W. Li , T. Wu , Q. Zhang , Y. Tang , M. Shao , H. Wang , Small 2022, 18, 2104148. 10.1002/smll.202104148 34766709

[23] X. Zeng , J. Mao , J. Hao , J. Liu , S. Liu , Z. Wang , Y. Wang , S. Zhang , T. Zheng , J. Liu , P. Rao , Z. Guo , Adv. Mater. 2021, 33, 2007416. 10.1002/adma.202007416 33576130

[24] L. Zhang , J. Xiao , X. Xiao , W. Xin , Y. Geng , Z. Yan , Z. Zhu , eScience 2023, 100205.

[25] J. Cao , Y. Sun , D. Zhang , D. Luo , L. Zhang , R. Chanajaree , J. Qin , X. Yang , J. Lu , Adv. Energy Mater. 2023, 14, 2302770.

[26] M. Fu , Q. Zhao , K. Long , Q. Li , G. Kuang , L. Zhou , W. Wei , X. Ji , L. Chen , Y. Chen , Adv. Funct. Mater. 2024, 34, 2311680.

