# Supplementary material for: Superfast Zincophilic Ion Conductor Enables Rapid Interfacial Desolvation Kinetics for Low‐Temperature Zinc Metal Batteries
Source: Adv Sci (Weinh). 2024 May 9;11(28):2401629. doi: 10.1002/advs.202401629 (PMC11267323; doi:10.1002/advs.202401629)
Supplement: Supplementary file 1 — Supporting Information [file ADVS-11-2401629-s001.pdf]

ADVANCED SCIENCE
Open Access

Supporting Information

for *Adv. Sci.*, DOI 10.1002/advs.202401629

Superfast Zincophilic Ion Conductor Enables Rapid Interfacial Desolvation Kinetics for Low-Temperature Zinc Metal Batteries

Xiaomin Cheng, Yinze Zuo, Yongzheng Zhang, Xinyu Zhao, Lujie Jia, Jing Zhang, Xiang Li, Ziling Wu, Jian Wang* and Hongzhen Lin**

Supporting Information

Superfast Zincophilic Ion Conductor Enables Rapid Interfacial Desolvation Kinetics for Low-Temperature Zinc Metal Batteries

Xiaomin Cheng, Yinze Zuo, Yongzheng Zhang, Xinyu Zhao, Lujie Jia, Jing Zhang, Xiang Li, Ziling Wu, Jian Wang,* and Hongzhen Lin**

X. Cheng, X. Zhao, Dr. L. Jia, Dr. J. Wang, Prof. H. Lin

i-Lab & CAS Key Laboratory of Nanophotonic Materials and Devices, Suzhou Institute of Nano-Tech and Nano-Bionics, Chinese Academy of Sciences, Suzhou 215123, P.R. China

E-mail: jian.wang@kit.edu; hzlin2010@sinano.ac.cn

Dr. Y. Zuo

Institute of New Energy Materials and Engineering, College of Materials Science and Engineering, Fuzhou University, Fuzhou 350108, P. R. China

Dr. Y. Zhang, Dr. X. Li, Z. Wu

State Key Laboratory of Chemical Engineering, East China University of Science and Technology, Shanghai 200237, P.R. China

E-mail: zhangyongzheng@ecust.edu.cn

Prof. J. Zhang

School of Materials Science and Engineering, Xi'an University of Technology, Xi'an 710048, China

Dr. J. Wang

Helmholtz Institute Ulm (HIU), D89081 Ulm, Germany

Karlsruhe Institute of Technology (KIT), D76021 Karlsruhe, Germany

E-mail: jian.wang@kit.edu

Experimental Section

Preparation of layered zinc silicate nanosheets: LZS nanosheets were synthesized following previous procedures with slight modification.[1] In brief, zinc chloride (0.75 mmol), ammonia chloride (10 mmol) and sodium silicate (1.266 mmol) were dissolved in 50 mL deionized water and stirred for 30 min. The above solution was placed into an autoclave (70 mL) and reacted at 140 °C for 12 h. The product was rinsed with deionized water, centrifuged, and dried in an oven at 60 °C overnight.

Preparation of Zn@LZS anode: Before assembling, the metallic Zn foil with a thickness of 100 μm was polished and washed to completed remove the initial passivation layer, exposing the fresh shine surface. Zn@LZS anode were produced by simple spray coating method. Typically, 80 mg LZS was added in 100 mL ethanol and magnetically stirred for 30 min, and then the above dispersion was sprayed on Zn foil under a heating platform at 80 °C.

Preparation of V_2O_{5-x} and MnO_2 cathodes: V_2O_5 and MnO_2 are commercial vanadium pentoxide and manganese dioxide, respectively. V_2O_5 was heated at 400 °C for 2 hours under H_2/Ar the atmosphere to obtain V_2O_{5-x}. The work electrode was prepared by mixing the active materials with carbon black and polyvinylidene fluoride (PVDF) in a mass ratio of 7:2:1. The mixture is compressed onto a carbon film and the average areal loading of the composite material was around 1.2 mg cm^{-2}.

Fabrication of Zn/Zn symmetric cells and full cells: For symmetric cell, it was assembled with Zn foil, 2 M $ZnSO_4$ electrolyte and glass microfiber GF/B (Whatman) separator in CR2025 coin cells. For Zn//MnO_2 cells, 2M $ZnSO_4$ + 0.2M $MnSO_4$ were used as the electrolyte, glass microfiber GF/B (Whatman) with a diameter 18 mm as the separator, and zinc foil (thickness of 100 μm) with a diameter 15 mm as the anode. For Zn//V_2O_{5-x} cells, 3M $Zn(CF_3SO_3)_2$ was used as the electrolyte, glass microfiber GF/B (Whatman) with a diameter 18 mm as the separator, and zinc foil with a diameter 15 mm as the anode.

Electrochemical Measurements: All the galvanostatic charge/discharge measurements were worked on a battery testing instrument (Land CT2001A) at different current densities. Cyclic voltammetry (CV) was investigated between 0.2-1.6

V for V_2O_{5-x} (0.8-1.8 V for Zn//MnO_2) using a coin cell configuration on a VMP-3 electrochemical workstation. Electrochemical impedance spectroscopy (EIS) is measured in a frequency range of 100 kHz to 0.1 Hz at open circuit potential and an amplitude of 5 mV. Linear polarization curves were measured by a three-electrode configuration in 2 M $ZnSO_4$ solution with a voltage window from -1.1 V to -0.6 V at a scan rate of 2 mV s^{-1}, where the Zn@LZS or untreated Zn worked as working electrode, Pt plate as the counter electrode, and Ag/AgCl as the reference electrode. In-situ growth of Zn dendrites was measured by using a digital microscope.

Characterization: The morphology and element distribution were gained by a Field-emission scanning electron microscope (SEM, Hitachi Regulus 8230) and a F20 S-Twin field-emission transmission electron microscope (TEM, Tecnai G2). X-ray diffraction (XRD) was conducted on a Rigaku D/Max 2550 using Cu Kα radiation. The elemental contents were analyzed by inductively coupled plasma atomic emission spectrometer (ICP-AES). N_2 adsorption-desorption test of LZS was performed on Quadrasorb SI Analyzer at 77 K. The in-situ sum frequency generation (SFG) measurements with/without the bias voltage of 20 mV were performed on the commercial picosecond laser system, similar to our previous studies.[2] In the SFG set-ups, the IR pulse ranges from 3000 to 3600 cm^{-1} and visible light wavelength is set at 532 nm, which are directly shone on the electrode/solvent interface with/without LZS layer. Raman spectra (Horiba LabRAM ARAMIS) was used to distinguish two solvent structures at the electrolyte/anode interface. Time-of-flight secondary-ion mass spectrometry (TOF-SIMS) was utilized to measure the three-dimensional morphology and element distribution of cycled Zn@LZS.

Computational methods: The first-principles calculation was performed by means of the DFT theory with Vienna ab initio simulation package (VASP) code. The generalized gradient approximation (GGA) proposed by Perdew-Burke-Ernzerhof and a cutoff energy of 450 eV for planewave basis set were adopted. A 2×2×1 Monkhorst Pack k grid was used for sampling the Brillouin zones at structure calculation, whereas a denser mesh of 5×5×1 was used for the electronic structure calculations. The Grimme's DFT-D3 scheme of dispersion correction was adopted to describe the van

der Waals interactions in these systems. The energy barriers for Zn ion diffusion in the LZS layer were computed by the nudged elastic band method with 5 images. The geometric structures were optimized by the cluster model at the B3LYP method. And all the calculations were performed in an implicit solvent model to simulate the aqueous environment. The successive desolvation energy under LZS cluster model adsorption condition was calculated according to the following equation:

$$E_d=E\{[Zn(H_2O)_{n-1}^{2+}]\}+E(H_2O)-E\{[Zn(H_2O)_n^{2+}]\}$$

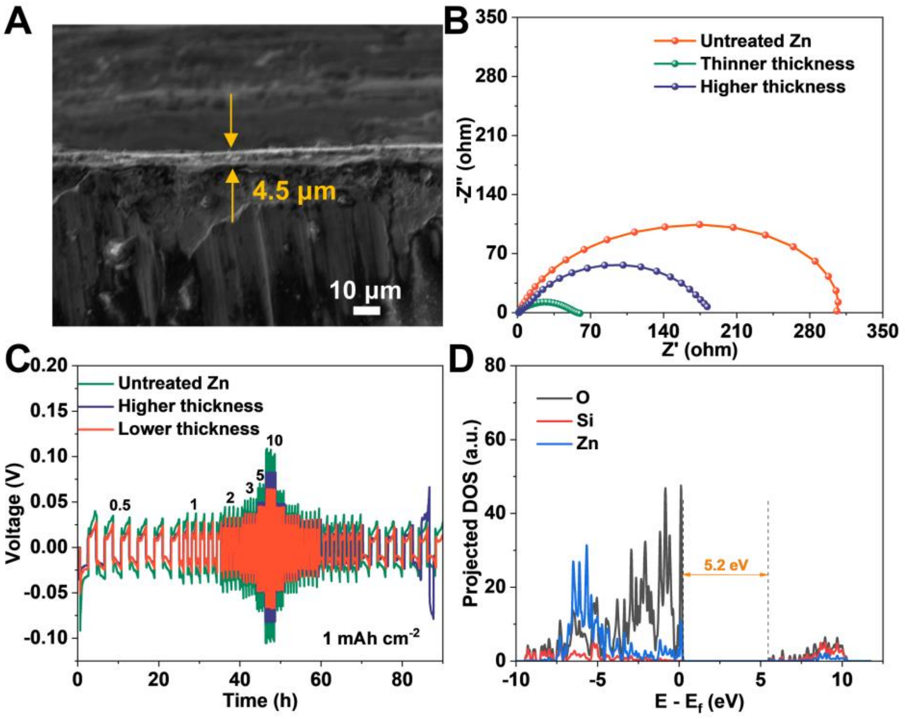

Figure S1. A) Cross-sectional SEM image of Zn@LZS with higher thickness. B) Nyquist plots of untreated Zn and Zn@LZS with different thickness. C) Rate capabilities during plating/stripping for 1 h. D) Partial density of states (PDOS) of LZS.

The thickness of the LZs layer would affect the electrochemical performance of Zn ion transport. In this work, a thickness of 700 nm was fabricated on the surface of Zn@LZS anode to probe the effect of LZS on interfacial desolvation and Zn^{2+} diffusion, the Zn@LZS anode with higher thickness of 4.5 μm was also fabricated. As shown in Figure S1, the Zn@LZS anode with higher thickness exhibits higher charge transfer impedance (R_{ct}) than the Zn@LZS anode with thinner layer. In addition, the symmetric cell with higher thickness of LZS layer exhibits higher overpotential and experiences a short circuit after rate cyclability within 80 h, which is attributed to the electrical insulation property and the sluggish Zn^{2+} transport rate.

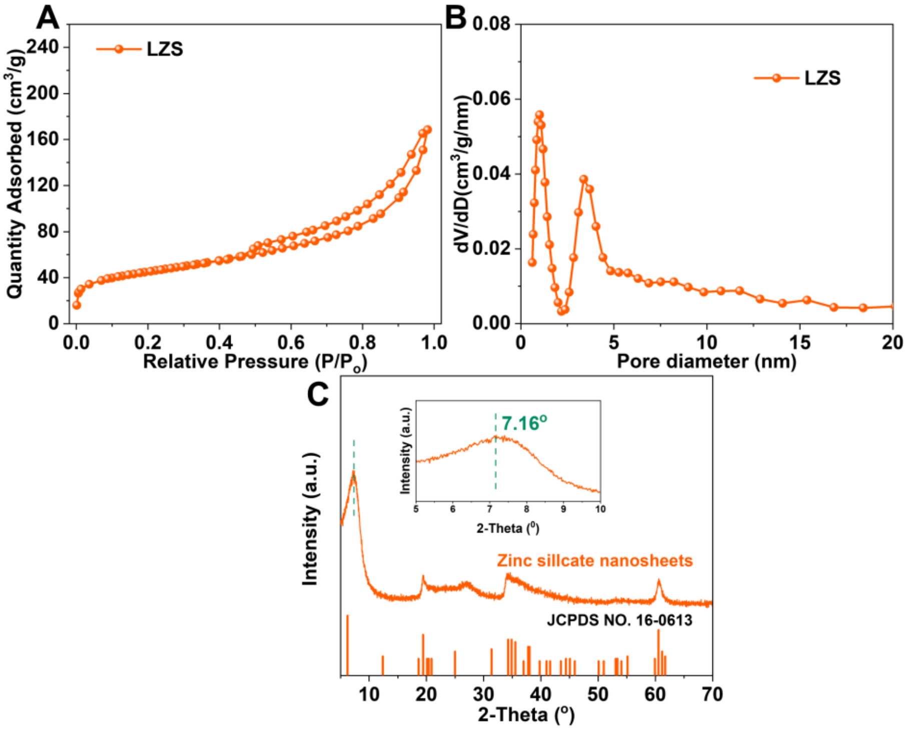

Figure S2. A) Nitrogen adsorption/desorption isotherm, B) pore-size distribution and C) XRD pattern of the synthesized LZS.

Very broad XRD peaks in the range of 5-70° can be indexed to the vermiculite structure of zinc silicate. LZS crystallizes in a monoclinic crystal system with a space group of *C2/m*.

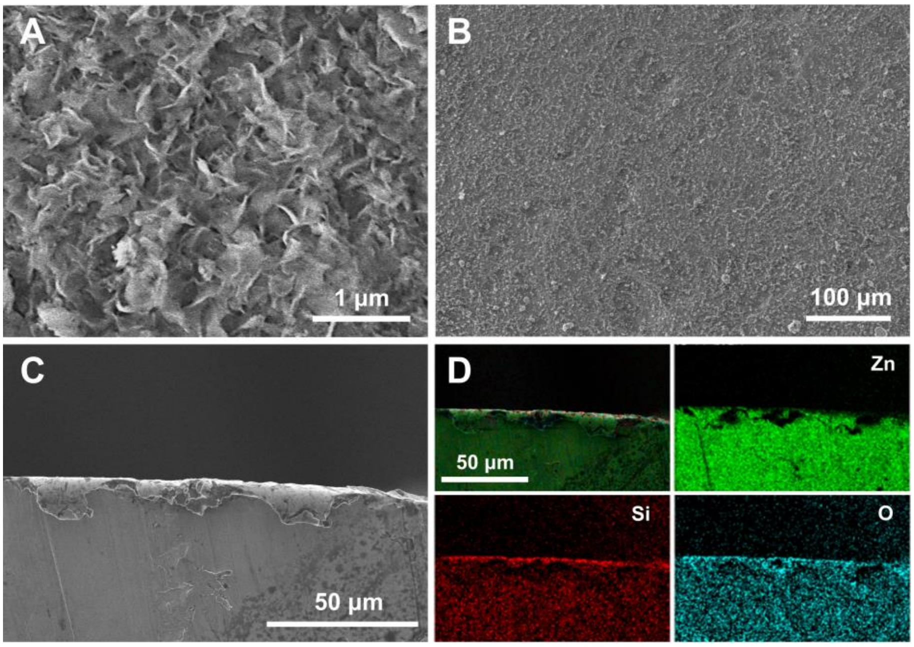

Figure S3. SEM images of synthesized LZS A) and the Zn@LZS anode: B) top view, C) side view. D) EDS mappings of cross-sectional of Zn@LZS anode.

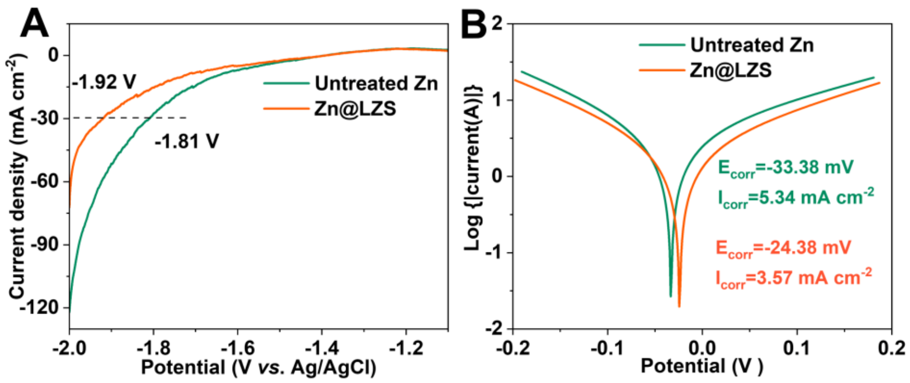

Figure S4. A) LSV and B) Tafel curves of untreated Zn and Zn@LZS anode.

Liner sweep voltammetry (LSV) and Tafel curves were employed to investigate the effect of LZS artificial layer on the anti-HER and anti-corrosion abilities. As shown in Figure S4, the overpotential of the Zn@LZS electrode (-1.92 V vs. SCE) is lower than that of the untreated Zn electrode (-1.81 V), demonstrating that the HER is partially alleviated due to unique LZS layer.[4] Compared with untreated Zn anode (-33.38 mV, 5.34 mA cm^{-2}), the increased corrosion potential (-24.38 mV) and the reduced corrosion current (3.57 mA cm^{-2}) of the Zn@LZS anode demonstrate the ability to withstand interfacial corrosion and mitigate the parasitic reactions.[5]

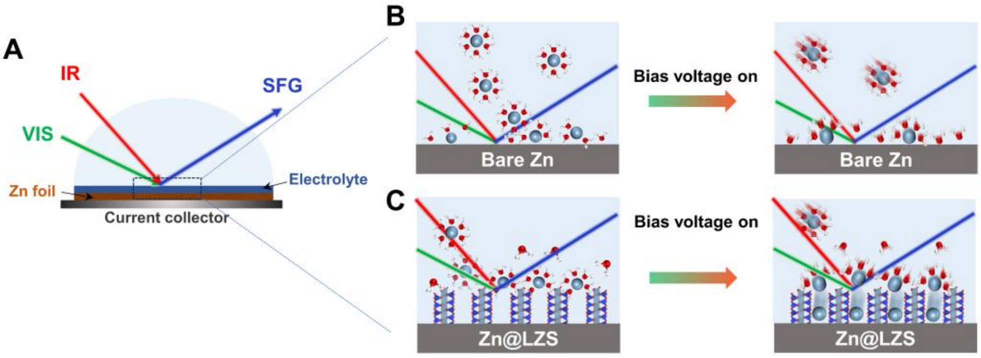

Figure S5. A) Schematic diagram of in situ SFG probing the electrode/electrolyte interface. The molecular states of the Zn^{2+} solvation structure in the B) untreated Zn/electrolyte and C) Zn@LZS/electrolyte interface under the conditions of open-circuit voltage state or bias voltage (20 mV).

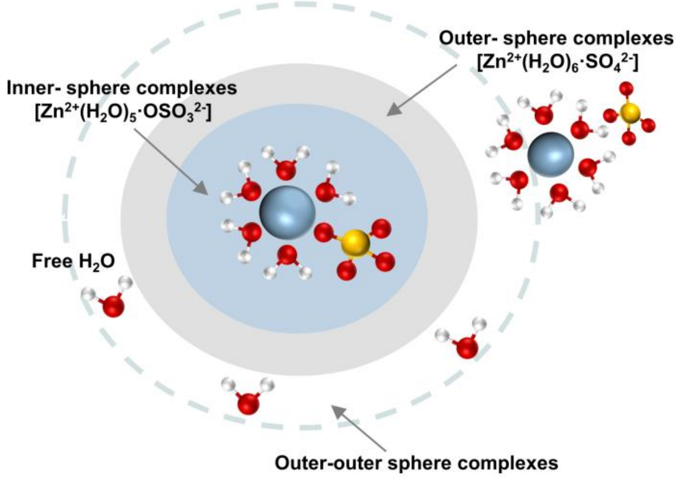

Figure S6. Schematic structure of $ZnSO_4$ aqueous solutions.

As displayed in Figure S6, the typical solvation shell structure consists of inner $[Zn(H_2O)_6]^{2+}$ species and corresponding outer shell species of SO_4^{2-}.

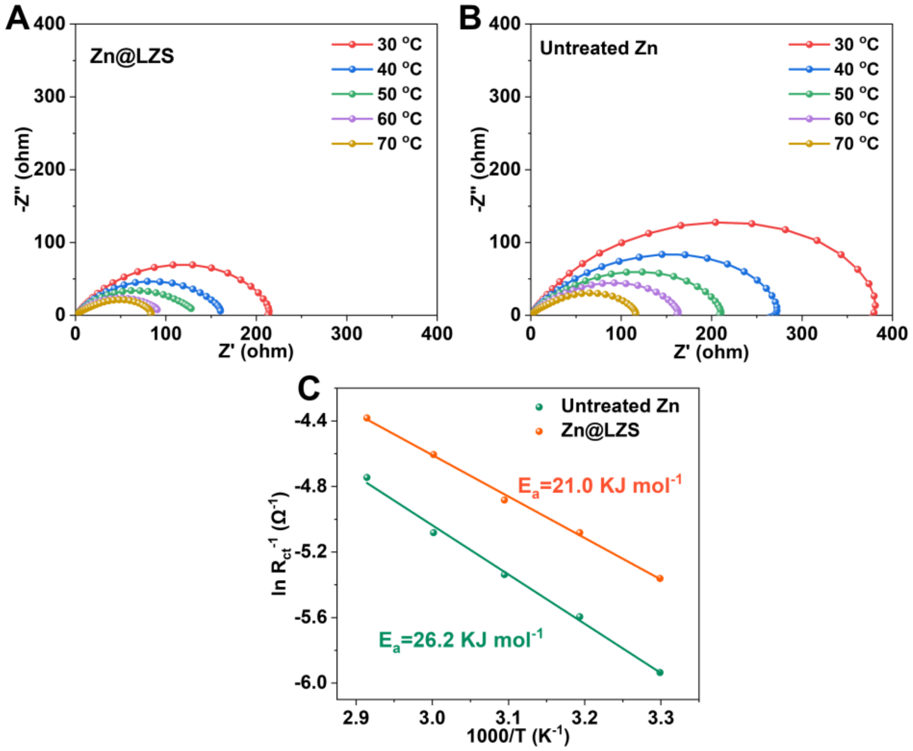

Figure S7. Nyquist plots of A) Zn@LZS and B) untreated Zn symmetric cells at various temperatures. C) Comparison of the activation energies with untreated Zn and Zn@LZS.

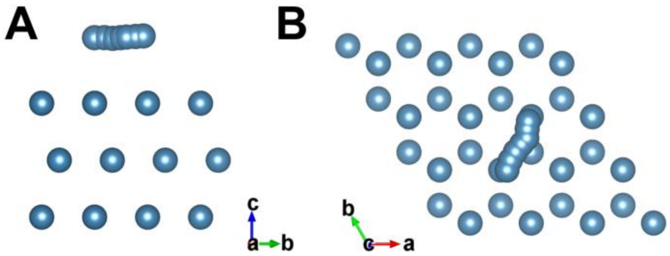

Figure S8. The possible migration pathway on untreated Zn.

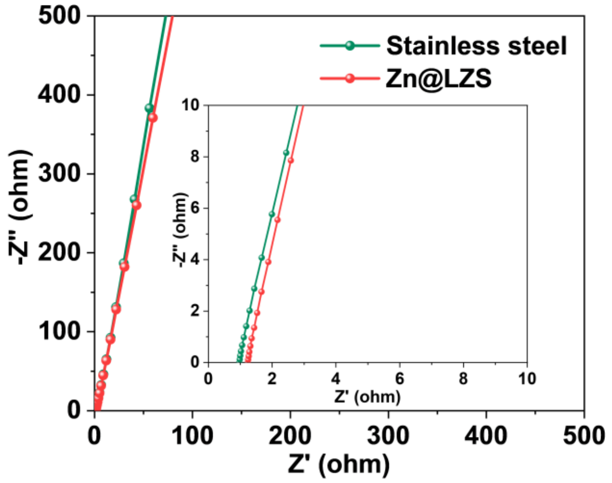

Figure S9. Ionic conductivity test of the Zn@LZS.

The ion conductivity (σ) was calculated by the following equation:

$$\sigma = \frac{L}{R_b S}$$

where L is the thickness of the ZSO coating, R_b represents the resistance of the coating layer and S is the effective contacting area.

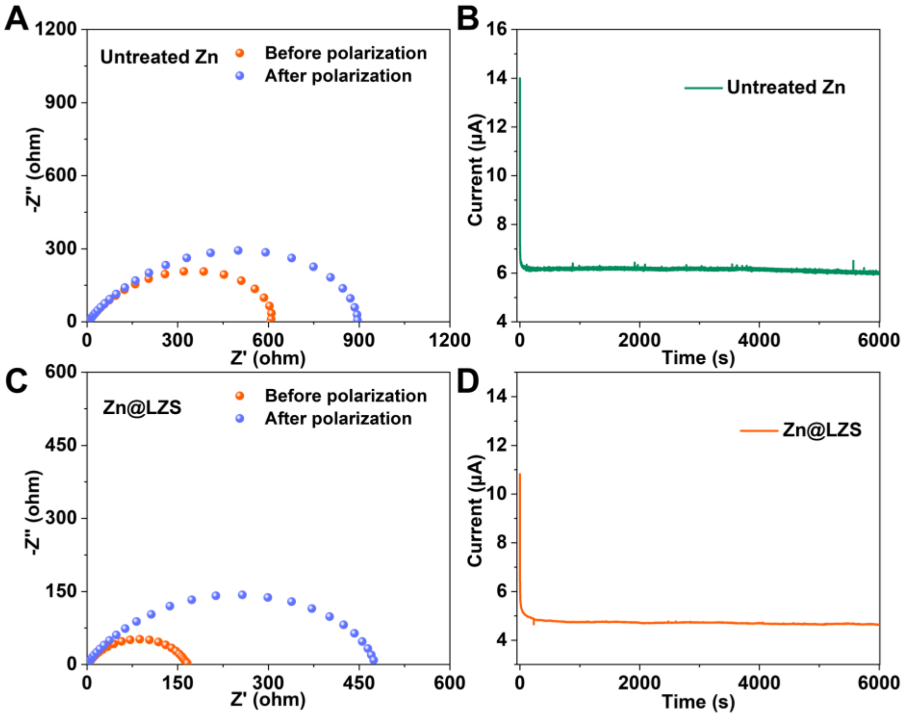

Figure S10. Nyquist profiles of A) untreated Zn and C) Zn@LZS symmetric cell before and after CA tests. CA curves of B) untreated Zn and D) Zn@LZS electrode at an over-potential of 20 mV.

Generally, the transference number ($t_{zn}{}^{2+}$) for both interfaces can be obtained based on the as-following equations:

$$t_{Zn^{2+}} = \frac{I_s(\Delta V - I_0 R_0)}{I_0(\Delta V - I_s R_s)}$$

where ΔV is the applied potential (20 mV); I_0 and R_0 are the initial current and interface resistance, respectively. I_S and R_S stand for the steady-state current and interface resistance, respectively.

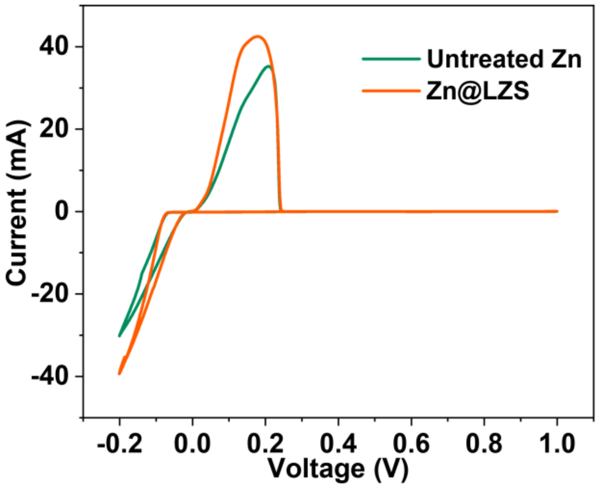

Figure S11. Cyclic voltammetry curves of untreated Zn//Cu cell and Zn@LZS cell.

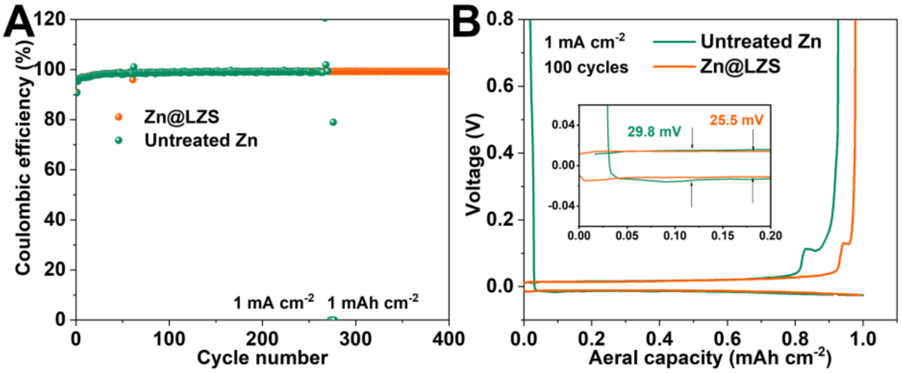

Figure S12. A) Coulombic efficiency and corresponding B) voltage/capacity plots of untreated Zn//Cu and Zn@LZS//Cu at 1 mA cm^{-2} after 100 cycles.

The voltage hysteresis of untreated Zn (29.8 mV) is higher than that of Zn@LZS (25.5 mV), suggesting a higher energy barrier for Zn nucleation/dissolution in the phase transition between Zn^{2+} and Zn metal. With the Zn plating and stripping continued, a slight voltage plateau emerged in the second half of the charging curve. Generally, this plateau is attributed to the dealloy process. The plating of Zn at Cu surface constructs Zn-Cu alloy, it dealloys when charging, resulting in such a voltage plateau in the charging curve.

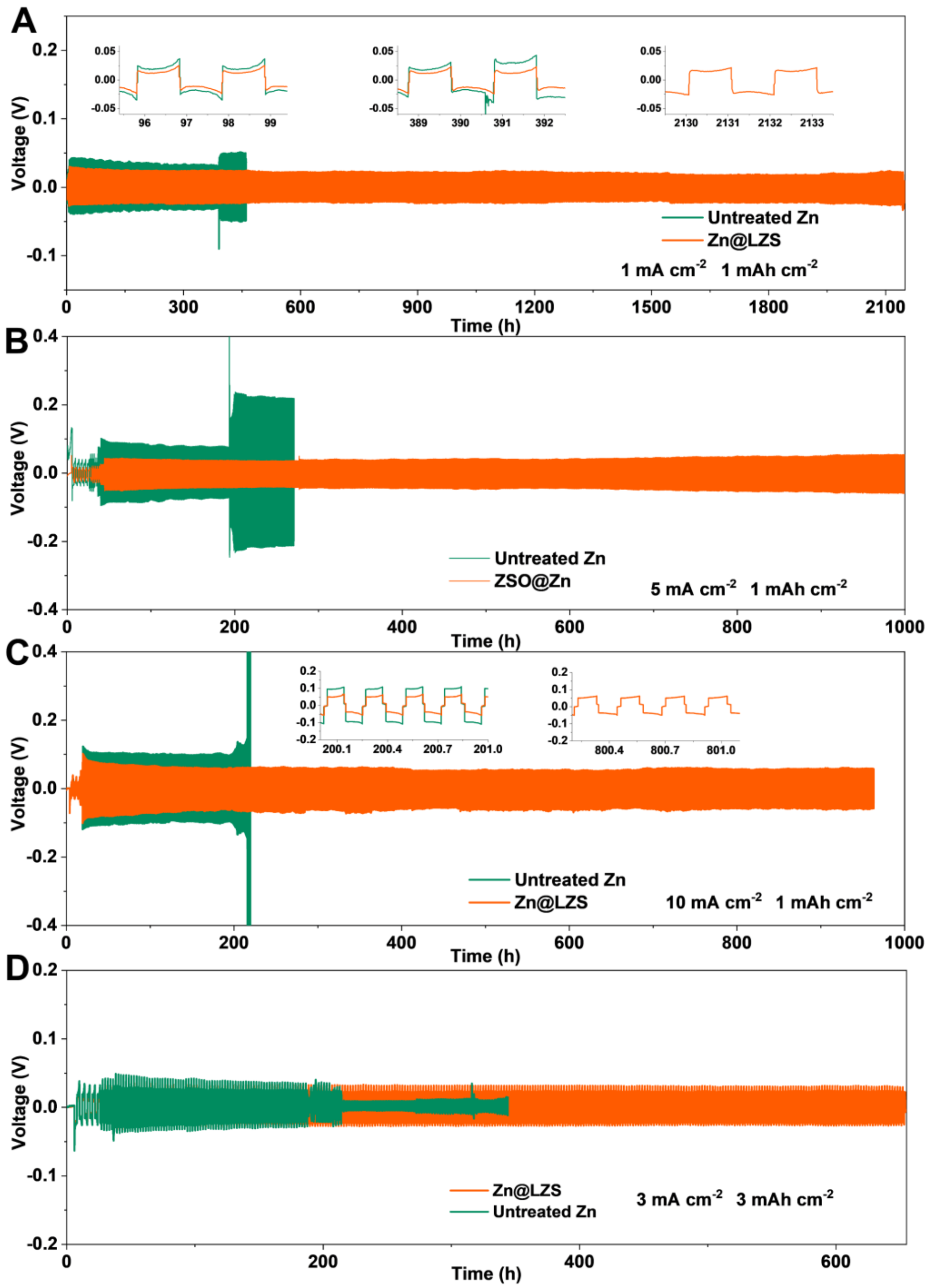

Figure S13. Long-term galvanostatic cycling performance of symmetrical untreated Zn and Zn@LZS cells at different test conditions: A) 1 mA cm^{-2} and 1 mAh cm^{-2}; B) 5 mA cm^{-2} and 1 mAh cm^{-2}; C) 10 mA cm^{-2} and 1 mAh cm^{-2}; D) 3 mA cm^{-2} and 3 mAh cm^{-2}.

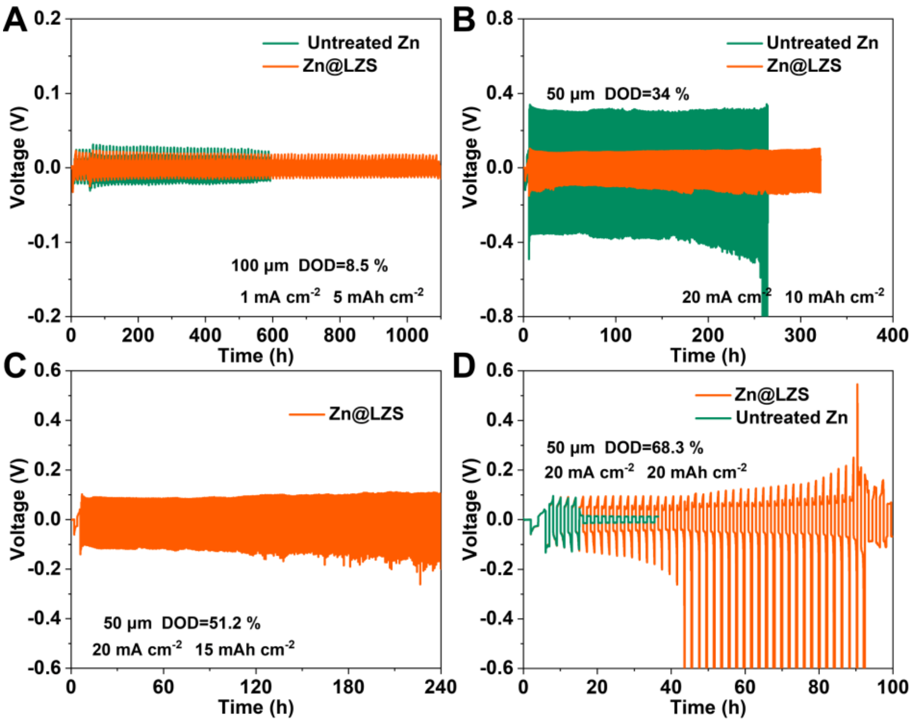

Figure S14. Long-term galvanostatic cycling of symmetrical Zn cells at high DOD: A) 8.5%; B) 34.0%; C) 51.2%; D) 68.3%.

The depth of discharge (DOD) of Zn anode is scientific metric to evaluate the ability of ZIBs in practical application, which was calculated using the following equation:

$$DOD = \frac{It}{mM} \times 100\%$$

Where I (mA) is the applied current density, t (h) is the discharge time, m (g) is the mass of the Zn in Zn anode, M (mA h g^{-1}) is the theoretical specific capacity of Zn (820 mAh g^{-1}).

As shown in Figure S14, the untreated Zn suffers from low DOD value and becomes rapid short circuit, which is ascribed to the imperfect Zn ion behaviors at the electrode/electrolyte interface. In contrast, the symmetrical cell with Zn@LZS shows a prolonged lifespan of 370 h and 240 h even under the super high areal capacity of severe DOD condition of 34% and 51.2% based on the metallic Zn, respectively.

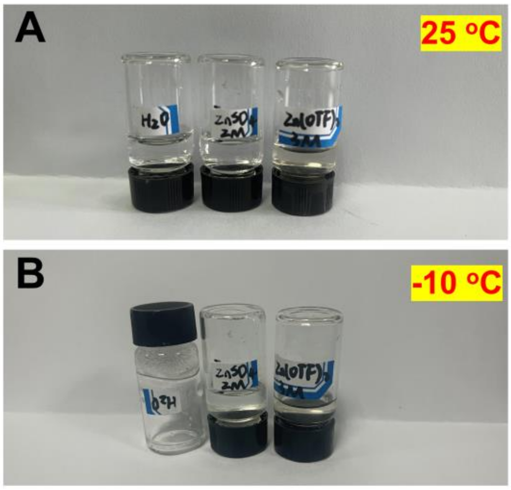

Figure S15. Optical photos of different electrolytes under A) 25 ºC and B) -10 ºC.

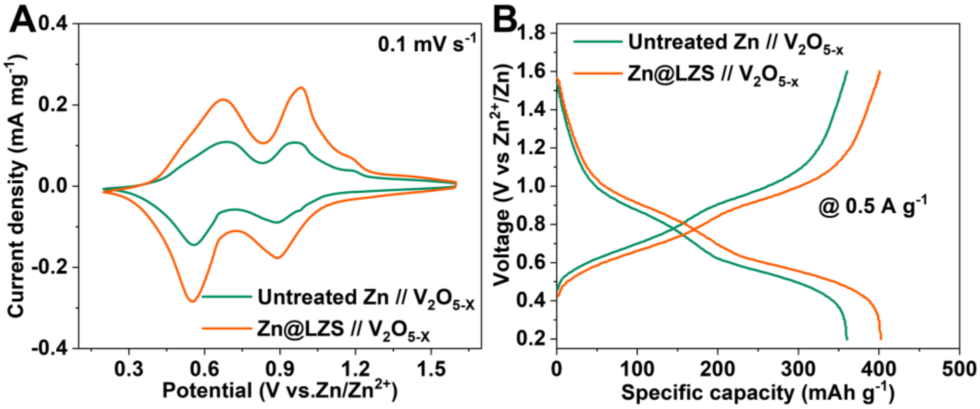

Figure S16. A) CV curves and B) galvanostatic charge/discharge curves V_2O_{5-x} cells.

Two distinct and typical pairs of redox peaks emerged in the cyclic voltammetry (CV), which are attributed to the redox pairs of V^{3+}/V^{4+} and V^{4+}/V^{5+}.[6] Noticeably, the full cell with LZS displays a higher intensity of peak current during the reduction/oxidation process compared to that of untreated Zn, indicating higher electrochemical activity owing to the effectiveness of the LZS layer.[7] In addition, the charge/diacharge curves detects two voltage plateaus at 0.56 V and 0.89 V, in accordance with the CV analysis.

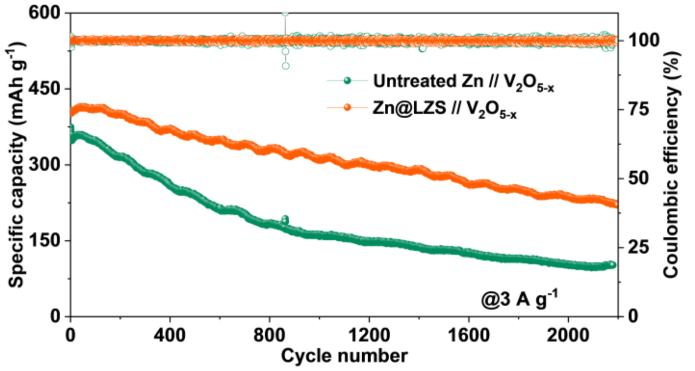

Figure S17. Galvanostatic cycling performance of V_2O_{5-x} cells at a current density of 3 A g^{-1}.

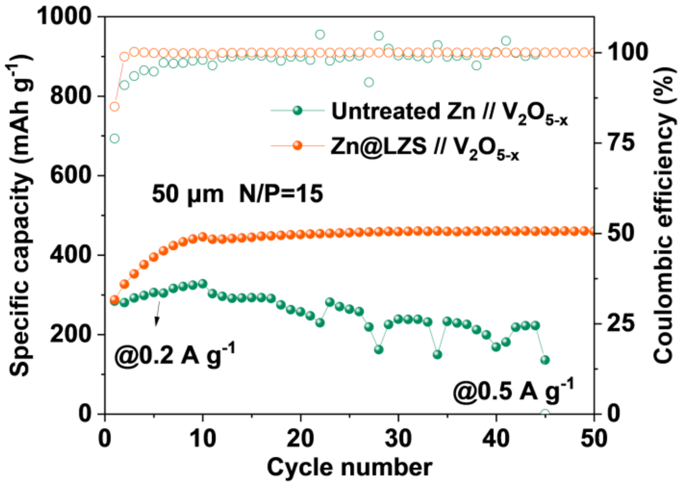

Figure S18. Cycling performance of Zn//V_2O_{5-x} full cells under lean electrolytes (30 μL mAh^{-1}) and 50 μm Zn foil.

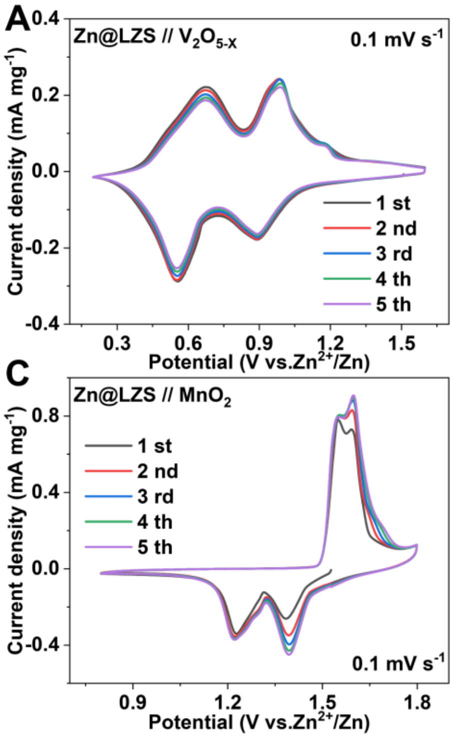

Figure S19. CV curves for A) Zn@LZS//V_2O_{5-x} and B) Zn//V_2O_{5-x} at the scan rate of 0.1 mV s^{-1}, CV curves for C) Zn@LZS//MnO_2 and D) Zn//MnO_2 at the scan rate of 0.1 mV s^{-1}.

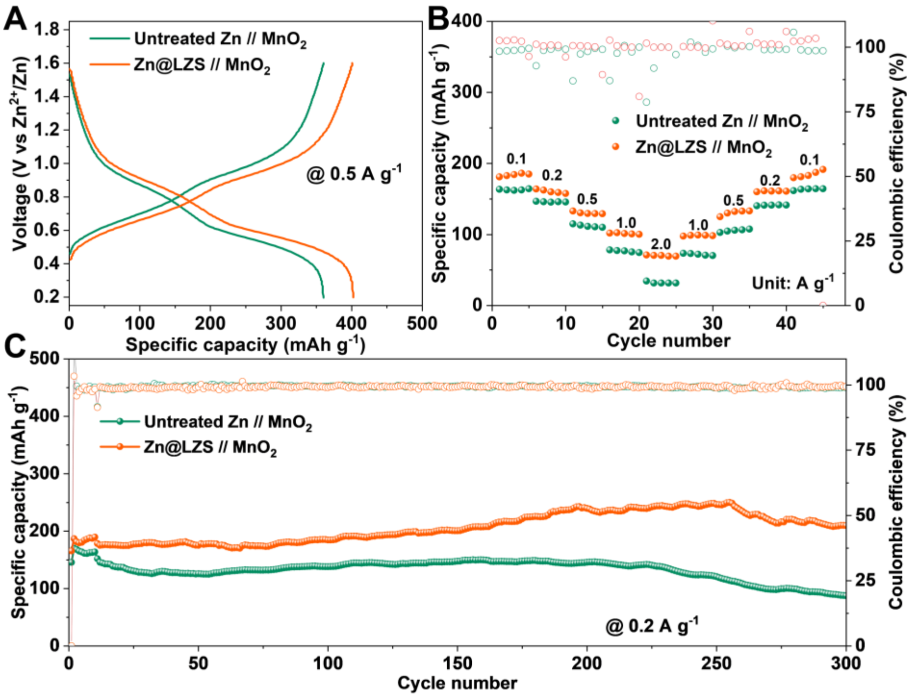

Figure S20. A) Galvanostatic charge/discharge curves and B) rate performance of Zn//MnO_2 cells. C) Galvanostatic cycling performance of Zn//MnO_2 cells at a current density of 0.2 A g^{-1}.

Such excellent electrochemical behavior further proved by assembled Zn//MnO_2 full cells. As displayed in Figure S20, Zn@LZS//MnO_2 full cell delivers an exccellent rate performance and good capacity stability.

Table S1. Unit cell parameters of LZS.

Unit cell parameters	Value (Å)
a	5.3
b	9.2
c	14.2
γ (deg)	96.8

Table S2. Comparison of the ionic conductivity of various coating with LZS (our work).

Coating	Ionic conductivity (mS cm^{-1})	Ref.
**LZS**	**2.16**	**Our work**
Perfluorosulfonic acid (PFSA) membranes	2.7×10^{-2}	[8]
Mg-Al LDH	5.24×10^{-2}	[9]
SnS	8.35×10^{-2}	[10]
$Zn_3(PO_4)_2$ and ZnF_2	1.403×10^{-2}	[11]
ZnP-NC	0.1207	[12]
P(VDF-TrFE) and $BaTiO_3$ (BTO/PVT@Zn)	0.65	[13]
NiCo-LDH	0.162	[14]
D-HfO_{2-X}	0.74	[15]
$Zn_3(OH)_2V_2O_7\cdot2H_2O$ (ZVO)	1.91	[16]
UiO-66-$(COOH)_2$	1.91	[17]
Zn-based Montmorillonite (MMT-Zn)	3.9	[18]

Table S3. Performance comparison of $Zn//V_2O_5$ full cells from previous researches with different modified Zn anodes and our work.

Anode	Cathode	Current Density ($A\ g^{-1}$)	Initial capacity ($mAh\ g^{-1}$)	Cycle number	Capacity retention rate (%)	Ref.
**Zn@LZS**	**Commercial V_2O_{5-x}**	**0.5**	**432.1**	**200**	**84.2**	**Our work**
ZIF-L@TM/Zn	Commercial V_2O_5	1	~320	200	71.8	[19]
Zn@CTF	Calcium-doped V_2O_5	1	~300	300	66.7	[20]
Sn@NHCF-Zn	Commercial V_2O_5	1	~250	200	60	[21]
PVDF-SBA15@ Zn	V_2O_5	1	~100	1000	82.1	[22]
CuZn@Zn	V_2O_5	1	~310	200	87	[23]
LLP@Treated Zn	V_2O_5	1	490	3850	45.5	[24]
rGO@ZnSi-Zn	V_2O_5	1	329	135	41	[18]
TCCF@Zn	V_2O_5	3	221.9	3000	53.4	[5]
TTP@Zn	V_2O_5	5	~150	6000	47	[25]
$(C_2F_4)_n$-C@Cu@Zn	V_2O_5	5	~145	2500	68.9	[26]
Zn-Sn-S	$NH_4V_4O_{10}$	1	248	150	80	[27]
SS-ZnP	$NH_4V_4O_{10}$	0.5	~290	170	53.4	[28]
Cu@AOF	$Zn_{0.5}VO_2$	1	~200	300	80	[29]

Reference

[1] J. Qu, C.-Y. Cao, Y.-L. Hong, C.-Q. Chen, P.-P. Zhu, W.-G. Song, Z.-Y. Wu, *J. Mater. Chem.* **2012**, 22, 3562-3567.

[2] a) J. Wang, J. Zhang, J. Wu, M. Huang, L. Jia, L. Li, Y. Zhang, H. Hu, F. Liu, Q. Guan, M. Liu, H. Adenusi, H. Lin, S. Passerini, *Adv. Mater.* **2023**, 35, 2302828; b) J. Wang, J. Yang, Q. Xiao, J. Zhang, T. Li, L. Jia, Z. Wang, S. Cheng, L. Li, M. Liu, H. Liu, H. Lin, Y. Zhang, *Adv. Funct. Mater.* **2020**, 31, 2007434.

[3] C. Deng, X. Xie, J. Han, Y. Tang, J. Gao, C. Liu, X. Shi, J. Zhou, S. Liang, *Adv. Funct. Mater.* **2020**, 30, 2000599.

[4] X. Cai, X. Wang, Z. Bie, Z. Jiao, Y. Li, W. Yan, H. J. Fan, W. Song, *Adv. Mater.* **2024**, 36, 2306734.

[5] P. Wu, L. Xu, X. Xiao, X. Ye, Y. Meng, S. Liu, *Adv. Mater.* **2023**, 36, 2306601.

[6] J. J. Ye, P. H. Li, H. R. Zhang, Z. Y. Song, T. Fan, W. Zhang, J. Tian, T. Huang, Y. Qian, Z. Hou, N. Shpigel, L. F. Chen, S. X. Dou, *Adv. Funct. Mater.* **2023**, 33, 2305659.

[7] S. Li, X. Xu, W. Chen, J. Zhao, K. Wang, J. Shen, X. Chen, X. Lu, X. Jiao, Y. Liu, Y. Bai, *Energy Storage Mater.* **2024**, 65, 103108.

[8] L. Hong, X. Wu, L.-Y. Wang, M. Zhong, P. Zhang, L. Jiang, W. Huang, Y. Wang, K.-X. Wang, J.-S. Chen, *ACS Nano* **2022**, 16, 6906-6915.

[9] Y. Yang, C. Liu, Z. Lv, H. Yang, X. Cheng, S. Zhang, M. Ye, Y. Zhang, L. Chen, J. Zhao, C. C. Li, *Energy Storage Mater.* **2021**, 41, 230-239.

[10] Y. Hu, C. Fu, S. Chai, Q. He, Y. Wang, M. Feng, Y. Zhang, A. Pan, *Advanced Powder Materials* **2023**, 2, 100093.

[11] Y. Chu, S. Zhang, S. Wu, Z. Hu, G. Cui, J. Luo, *Energy Environ. Sci.* **2021**, 14, 3609-3620.

[12] T. Wang, Q. Xi, Y. Li, H. Fu, Y. Hua, E. G. Shankar, A. K. Kakarla, J. S. Yu, *Adv. Sci.* **2022**, 9, 2200155.

[13] Q. Zong, B. Lv, C. Liu, Y. Yu, Q. Kang, D. Li, Z. Zhu, D. Tao, J. Zhang, J. Wang, Q. Zhang, G. Cao, *ACS Energy Lett.* **2023**, 8, 2886-2896.

[14] C. Ma, X. Wang, W. Lu, C. Wang, H. Yue, G. Sun, D. Zhang, F. Du, *Chem. Eng. J.* **2022**, 429, 132576.

[15] K. Zhang, C. Li, J. Liu, S. Zhang, M. Wang, L. Wang, *Small* **2024,** 20, 2306406.

[16] Y. Zhou, G. Li, S. Feng, H. Qin, Q. Wang, F. Shen, P. Liu, Y. Huang, H. He, *Adv Sci.* **2023**, 10, 2205874.

[17] W. Xin, J. Xiao, J. Li, L. Zhang, H. Peng, Z. Yan, Z. Zhu, *Energy Storage Mater.* **2023**, 56, 76-86.

[18] X. Dong, Y. Peng, Y. Wang, H. Wang, C. Jiang, C. Huang, C. Meng, Y. Zhang, *Energy Storage Mater.* **2023**, 62, 102937.

[19] Y. Tao, S. W. Zuo, S. H. Xiao, P. X. Sun, N. W. Li, J. S. Chen, H. B. Zhang, L. Yu, *Small* **2022**, 18, 2203231.

[20] G. Li, X. Wang, S. Lv, J. Wang, X. Dong, D. Liu, *Chem. Eng. J.* **2022**, 450, 138116.

[21] H. Yu, Y. X. Zeng, N. W. Li, D. Luan, L. Yu, X. W. Lou, *Sci. Adv.* **2022**, 8, eabm5766.

[22] M.-C. Liu, C.-Y. Tian, D.-T. Zhang, Y.-S. Zhang, B.-M. Zhang, Y.-Y. Wang, C.-Y. Li, M.-J. Liu, B. Gu, K. Zhao, L.-B. Kong, Y.-L. Chueh, *Nano Energy* **2022**, 103, 107805.

[23] B. Li, K. Yang, J. Ma, P. Shi, L. Chen, C. Chen, X. Hong, X. Cheng, M. C. Tang, Y. B. He, F. Kang, *Angew. Chem. Int. Ed.* **2022**, 61, e202212587.

[24] Z. Na, H. Qi, S. Li, Y. Wu, Q. Wang, G. Huang, *ACS Energy Lett.* **2023**, 8, 3297-3306.

[25] X. Qi, F. Xie, Y.-T. Xu, H. Xu, C.-L. Sun, S.-H. Wang, J.-Y. Hu, X.-F. Wang, *Chem. Eng. J.* **2023**, 453, 139963.

[26] Q. Li, H. Wang, H. Yu, M. Fu, W. Liu, Q. Zhao, S. Huang, L. Zhou, W. Wei, X. Ji, Y. Chen, L. Chen, *Adv. Funct. Mater.* **2023**, 33, 2303466.

[27] Z. Yang, Q. Zhang, W. Li, C. Xie, T. Wu, C. Hu, Y. Tang, H. Wang, *Angew. Chem. Int. Ed.* **2022**, 62, e202215306.

[28] C. Cao, K. Zhou, W. Du, C. C. Li, M. Ye, Y. Zhang, Y. Tang, X. Liu, *Adv. Energy Mater.* **2023**, 13, 2301835.

[29] C. Wang, D. Wang, D. Lv, H. Peng, X. Song, J. Yang, Y. Qian, *Adv. Energy Mater.*

2023, 13, 2204388.
